# A racemic formal total synthesis of clavukerin A using gold(I)-catalyzed cycloisomerization of 3-methoxy-1,6-enynes as the key strategy

**DOI:** 10.3762/bjoc.7.84

**Published:** 2011-06-01

**Authors:** Jae Youp Cheong, Young Ho Rhee

**Affiliations:** 1 Department of Chemistry, POSTECH (Pohang University of Science and Technology), Hyoja-dong san 31, Nam-gu, Pohang, Kyungbook, Republic of Korea 790-784

**Keywords:** clavukerin A, cycloisomerization, gold catalyst, hydrogenation, stereoselectivity

## Abstract

An efficient formal total synthesis of (±)-clavukerin A was accomplished via a gold-catalyzed cycloisomerization of a 3-methoxy-1,6-enyne **5** as the key strategy followed by Rh-catalyzed stereoselective hydrogenation of the cycloheptenone **4**.

## Findings

Clavukerin A is a member of marine trinorguaiane sesquiterpene natural products. It was first isolated in 1983, by the group of Kitawara, from the Okinawa soft coral *Clavularia koellikeri*. The structure of clavukerin A was established by CD spectra and X-ray diffraction [1]. The first total synthesis of clavukerin A was reported by Asaoka in 1991, which was followed by several other racemic and enantioselective syntheses [2–14]. Herein, we report a short formal total synthesis of racemic clavukerin A employing the gold(I)-catalyzed cycloisomerization of a 3-methoxy-1,6-enyne as the key strategy, which was recently developed by us [15]. This reaction provides cycloheptane frameworks in a unique manner and illustrates the utility of the gold-catalyzed reactions [16–23].

**Scheme 1:**
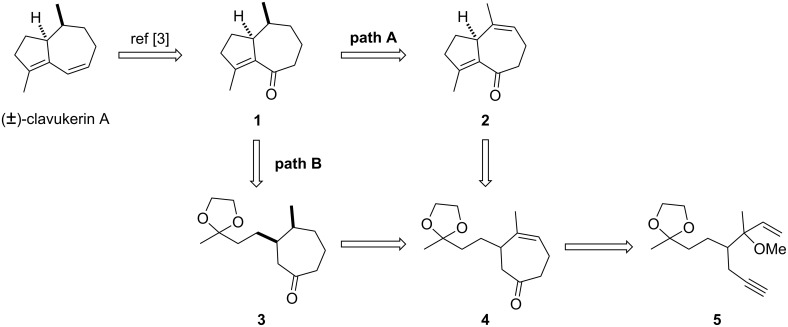
Retrosynthetic analysis.

From a retrosynthetic point of view, we envisioned two different approaches to the key enone intermediate **1** [3] to clavukerin A, starting from the cycloheptenone **4** (Scheme 1). In the first approach, enone **1** could be prepared by the sequential cyclization and the chemo- and stereoselective hydrogenation from cycloheptenone **4** (path A). Alternatively, enone **1** could be accessed by the hydrogenation of **4** and the subsequent cyclization (path B). The cycloheptenone **4** could then be synthesized from the enyne substrate **5** by gold(I)-catalyzed cycloisomerization.

**Scheme 2:**
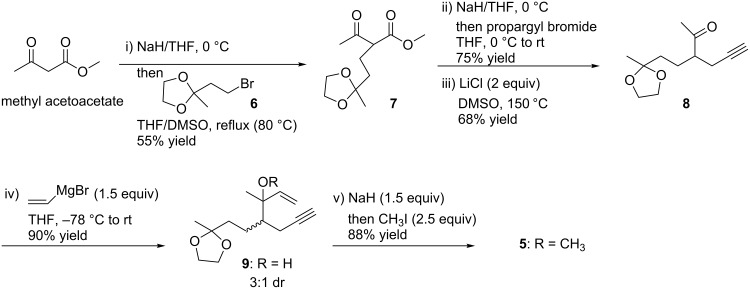
Preparation of compound **5**.

The synthesis of enyne substrate **5** commenced with the alkylation of methyl acetoacetate with the known bromide **6** [24] to provide compound **7** in 55% yield (Scheme 2). Propargylation of **7** followed by the decarbomethoxylation with LiCl [25] gave the ketone **8** in 51% yield (over two steps). Addition of the vinyl group to this ketone gave the alkynol **9** in 90% yield as an inseparable 3:1 mixture of diastereomers. The diastereomeric ratio was determined by integration of the ^1^H NMR spectrum of the crude reaction product. Subsequent methylation gave the 1,6-enyne **5** in 88% yield.

**Scheme 3:**
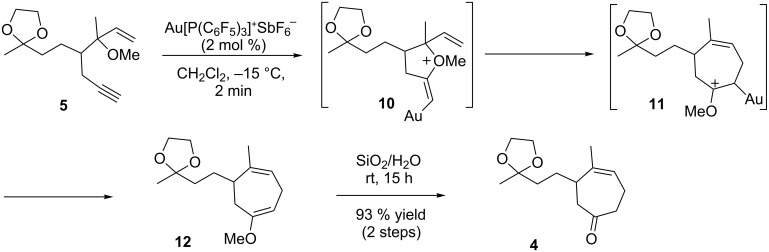
Synthesis of the cycloheptenone **4**.

We then investigated the gold-catalyzed cycloisomerization of enyne **5** using the optimized conditions from our previous study [15]. The use of the pre-generated complex Au[P(C_6_F_5_)_3_]^+^SbF_6_^−^ (2 mol %) provided the relatively unstable enol ether **12**, which was then immediately treated with aqueous silica gel to give the ketone **4** in 93% yield over two steps. Formation of **12** was unambiguously confirmed by the analysis of ^1^H NMR data of the crude reaction mixture. From a mechanistic viewpoint, the reaction presumably proceeds via the initial heterocyclization intermediate **10** and the subsequently rearranged intermediate **11** (Scheme 3). Notably, when the gold(I)-catalyzed reaction was carried out on a multi-mmol scale, there was no decrease in the yield at the same catalyst loading.

**Scheme 4:**
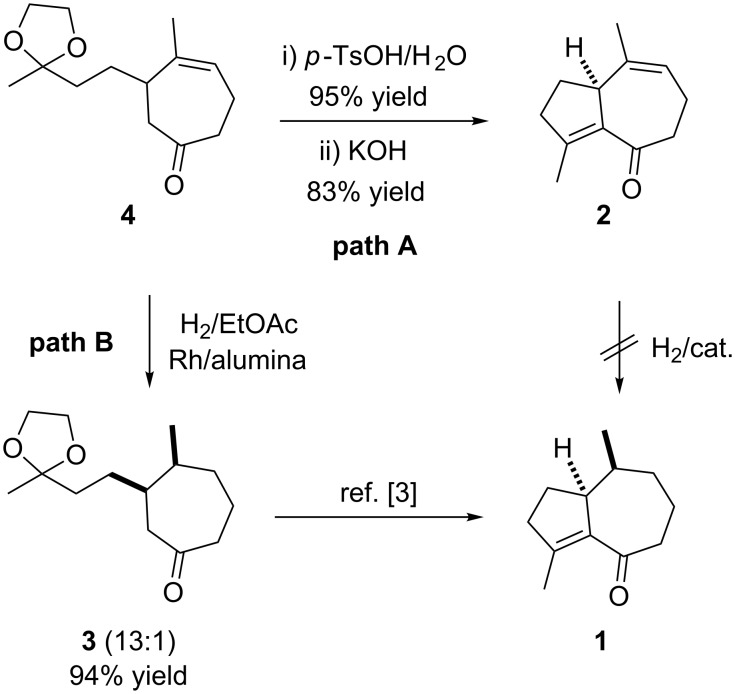
Completion of the formal synthesis of clavukerin A.

With ketone **4** in hand, the final stage in the formal synthesis of clavukerin A was explored. We first investigated the cyclization–hydrogenation strategy (path A in Scheme 4). Deprotection of **4** and the aldol condensation of the resulting diketone under basic conditions proceeded smoothly to give the enone **2** in good yield. However, extensive attempts at the chemoselective hydrogenation of the trisubstituted olefin **2** gave only compound **1** with poor selectivity. For example, various metal (Pd or Rh)-catalyzed hydrogenations resulted in a mixture of **1** and **3**. This problem was also noted in another work on the synthesis of clavukerin A [13].

Thus, we decided to investigate the alternative strategy that involved sequential hydrogenation–cyclization of **4**. Initial efforts using various Pd catalysts or Wilkinson catalyst again showed poor stereoselectivity for the hydrogenation. However, with a Rh/alumina catalyst the selectivity was significantly improved and afforded the *cis*-ketone **3** in 94% yield with ~13:1 selectivity. The structure of **3** was unambiguously confirmed by comparison of the ^1^H and ^13^C data with those in the literature [3]. Because the ketone **3** was previously transformed into the enone **1** [3], synthesis of **3** represents the completion of the formal synthesis of clavukerin A.

In summary, a formal synthesis of racemic clavukerin A was accomplished via the gold(I)-catalyzed cycloisomerization of a 3-methoxy-1,6-enyne as the key strategy and stereoselective Rh-catalyzed hydrogenation. Notably, the gold(I)-catalyzed reaction was compatible with the acid-sensitive functional group. Further application of the gold(I)-catalyzed cycloisomerization reaction of 3-methoxy-1,6-enynes to the enantioselective synthesis of more structurally complex cycloheptane natural products is in progress, and will be reported in due course.

## Supporting Information

File 1 Experimental section for the preparation of compounds **2**–**12**, and ^1^H and ^13^C NMR spectra for all new compounds.
