# Supplementary material for: A racemic formal total synthesis of clavukerin A using gold(I)-catalyzed cycloisomerization of 3-methoxy-1,6-enynes as the key strategy
Source: Beilstein J Org Chem. 2011 Jun 1;7:740–3. doi: 10.3762/bjoc.7.84 (PMC3135252; doi:10.3762/bjoc.7.84)
Supplement: File 1 — Experimental section for the preparation of compounds 2–12, and 1H and 13C NMR spectra for all new compounds. [file Beilstein_J_Org_Chem-07-740-s001.pdf]

Supporting Information

for

A racemic formal total synthesis of clavukerin A using gold(I)-catalyzed cycloisomerization of 3-methoxy-1,6-enynes as the key strategy

Jae Youp Cheong and Young Ho Rhee*

Address: Department of Chemistry, POSTECH (Pohang University of Science and Technology), Hyoja-dong san 31, Nam-gu, Pohang, Kyungbook, Republic of Korea 790-784

Email: Young Ho Rhee* - yhrhee@postech.ac.kr

* Corresponding author

Experimental section for the preparation of compounds **2**–**12**, and ^{1}H and ^{13}C NMR spectra for all new compounds.

1. General Information

All commercially available chemicals were used without further purification. All solvents were dried and distilled according to the standard methods before use. Au[P(C_6F_5)$_3$]Cl was prepared according to the literature procedure [1] Experiments were performed in flame-dried glassware with rubber septa under a positive pressure of nitrogen. Reactions were monitored by thin-layer chromatography carried out on 0.25 mm E. Merck silica gel plates (60F-254) using UV light as a visualizing agent and acidic *p*-anisaldehyde, PMA/EtOH and heat as the developing agent. Flash chromatography was carried out on Merck 60 silica gel (230-400 mesh). ^{1}H and ^{13}C NMR spectra were recorded with a Bruker (300 MHz) spectrometer. ^{1}H NMR spectra were referenced to $CDCl_3$ (7.26 ppm) and reported as follows; chemical shift, multiplicity (s = singlet, d = doublet, t = triplet, m = multiplet, dd = doublet of doublets, td = triplet of doublets, ddd = doublet of doublet of doublets). Chemical shifts of the ^{13}C NMR spectra were measured relative to $CDCl_3$ (77.23 ppm). Mass spectral data were obtained from the Korea Basic Science Institute (Daegu) on a Jeol JMS 700 high resolution mass spectrometer.

2. Representative procedure

Compound 7

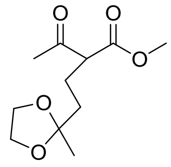

7

The literature procedure [2] was modified. A solution of methyl acetoacetate (5.29 g, 4.92 mL, 45.6 mmol) in THF (8.0 mL) was slowly added at 0 °C to a suspension of sodium hydride (60% in mineral oil, 1.82 g, 45.4 mmol) in THF (30 mL), followed by the addition of DMSO (15 mL). After stirring for 30 min at rt, a solution of 2-(2-bromoethyl)-2-methyl-1,3-dioxolane (8.00 g, 41.0 mmol) in THF (7.0 mL) was added, followed by the addition of DMSO (7.0 mL). The reaction mixture was heated under reflux (80 °C) for 15 h. The reaction mixture was cooled to 0 °C and quenched with water. THF was removed under reduced pressure. The crude product was extracted with diethyl ether (100 mL × 3) and the extracts washed with sat. NH_4Cl solution (100 mL × 2). The organic layers were combined, dried over Na_2SO_4, and concentrated. The residue was purified by flash chromatography on silica gel (eluent hexane:ethyl acetate = 85:15) to give the compound **7** as a colorless oil (5.20 g, 22.6 mmol, 55% yield, 17% of 2-(2-bromoethyl)-2-methyl-1,3-dioxolane was recovered). R_f =

0.26 (hexane:ethyl acetate = 70:30); ^{1}H NMR (300 MHz, $CDCl_3$): δ = 1.30 (s, 3H), 1.59−1.66 (m, 2H), 1.91−2.03 (m, 2H), 2.22 (s, 3H), 3.48 (t, J = 7.4 Hz, 1H), 3.72 (s, 3H), 3.87−3.97 (m, 4H); ^{13}C NMR (75 MHz, $CDCl_3$): δ = 22.8, 23.9, 29.1, 36.4, 52.5, 59.4, 64.8, 109.6, 170.3, 203.2; IR: (cm^{-1}) ν 2983, 2955, 2885, 1744, 1717, 1436, 1378, 1360, 1219, 1147, 1062; HRMS calcd for $C_{11}H_{19}O_5$: 231.1232. found: 231.1231.

Compound 8

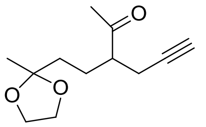

8

A solution of compound **7** (2.65 g, 11.5 mmol) in THF (5.0 mL) was slowly added. to a suspension of sodium hydride (60% in mineral oil, 553 mg, 13.8 mmol) in THF (24 mL) at 0 °C. After stirring for 30 min at rt, propargyl bromide (80% solution in toluene, 1.54 mL, 13.8 mmol) was added. The reaction mixture was stirred for 3 h, cooled to 0 °C and the reaction quenched with water. THF was removed under reduced pressure. The crude product was extracted with diethyl ether (50 mL × 3) and the extracts washed with sat. NH_4Cl solution (50 mL × 3). The organic layers were combined, dried over Na_2SO_4, and concentrated. The residue was purified by flash chromatography on silica gel (eluent hexane:ethyl acetate = 90:10) to give the propargylated product as a white solid (2.31 g, 8.61 mmol, 75% yield, m.p = 62−64 °C). R_f = 0.31 (hexane:ethyl acetate = 70:30); ^{1}H NMR (300 MHz, $CDCl_3$): δ = 1.30 (s, 3H), 1.38 (dd, J = 12.7 Hz, J = 4.3 Hz, 1H), 1.51 (td, J = 12.8 Hz, J = 5.1 Hz, 1H), 1.99 (t, J = 2.6, 1H), 2.03−2.13 (m, 1H), 2.16 (s, 3H), 2.18−2.27 (m, 1H), 2.70−2.71 (m, 1H), 3.73 (s, 3H), 3.90−3.94 (m, 4H); The product (2.62 g, 9.77 mmol) and LiCl (828 mg, 19.5) were dissolved in DMSO (13 mL) and water (0.5 mL). The reaction mixture was heated to 150 °C for 15 h then cooled to 0 °C and the reaction quenched with water. The crude product was extracted with ether (100 mL × 3) and the extracts washed with water (100 mL × 3). The organic layers were combined, dried over Na_2SO_4, and concentrated. The residue was purified by flash chromatography on silica gel (eluent hexane:ethyl acetate = 90:10) to give compound **8** as a colorless oil (1.40 g, 6.66 mmol, 68% yield). R_f = 0.47 (hexane:ethyl acetate = 70:30); ^{1}H NMR (300 MHz, $CDCl_3$): δ = 1.29 (s, 3H), 1.53−1.73 (m, 3H), 1.75−1.87 (m, 1H), 1.98 (t, J = 2.7 Hz, 1H), 2.20 (s, 3H), 2.35 (ddd, J = 16.9 Hz, J = 6.5 Hz, J = 2.7 Hz, 1H), 2.42 (ddd, J = 16.9 Hz, J = 7.4 Hz, J = 2.7 Hz, 1H), 2.66−2.75 (m, 1H), 3.88−3.94 (m, 4H); ^{13}C NMR (75 MHz, $CDCl_3$): δ = 20.3, 23.9, 25.2, 29.7, 36.1, 51.2, 64.8, 64.9, 70.2, 81.7, 109.7, 210.4; IR: (cm^{-1}) ν 3281, 2956, 1713, 1378, 1224, 1168, 1060; HRMS calcd for $C_{12}H_{19}O_3$(FAB+): 211.1334. found: 211.1336.

Compound 9

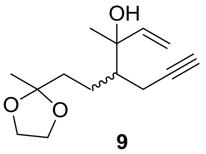

9

A solution of vinylmagnesium bromide (1.0 M sol. in THF, 2.4 mL, 2.4 mmol) was slowly added to a solution of compound **8** (340 mg, 1.62 mmol) in THF (8.0 mL) at -78 °C. The reaction mixture was allowed to warm to rt and stirred for 3 h. The reaction mixture was cooled to 0 °C and the reaction quenched with water. THF was removed under reduced pressure. The crude product was extracted with diethyl ether (50 mL × 3) and the extracts washed with sat. NH_4Cl solution (50 mL × 3). The organic layers were combined, dried over Na_2SO_4, and concentrated. The residue was purified by flash chromatography on silica gel (eluent hexane*:*ethyl acetate = 80:20) to give compound **9** as a colorless oil (345 mg, 1.45 mmol, 90% yield, dr = 3*:*1). R_f = 0.44 (hexane*:*ethyl acetate = 70:30); ^{1}H NMR (300 MHz, $CDCl_3$): δ = 1.27 (s, 3H), 1.32 (s, 3H), 1.50−1.65 (m, 3H), 1.68−1.76 (m, 1H), 1.81−1.92 (m, 1H), 2.00 (t, *J* = 2.7 Hz, 1H), 2.11 (s, 1H), 2.26−2.40 (m, 2H), 3.90−3.95 (m, 4H), 5.10 (dd, *J* = 10.7 Hz, *J* = 1.3 Hz, 1H), 5.27 (dd, *J* = 17.2 Hz, *J* = 1.3 Hz, 1H), 5.92 (dd, *J* = 17.3 Hz, *J* = 10.7 Hz, 1H); {distinctive signals for the minor} δ = 1.31 (s, 3H), 2.08 (s, 1H), 5.25 (dd, *J* = 10.7 Hz, *J* = 1.4 Hz, 1H), 5.88 (dd, *J* = 17.2 Hz, *J* = 10.8 Hz, 1H); ^{13}C NMR (75 MHz, $CDCl_3$): δ = 18.5, 18.6, 22.9, 23.8, 23.9, 25.6, 26.4, 37.7, 37.7, 46.2, 46.9, 64.7, 64.8, 70.3, 70.5, 76.0, 76.1, 83.6, 83.9, 110.3, 110.3, 112.6, 113.1, 143.7, 144.6; IR: (cm^{-1}) ν 3473, 3295, 2982, 2884, 1378, 1218, 1060; HRMS calcd for $C_{14}H_{23}O_3$(FAB+): 239.1647. found: 239.1650.

Compound 5

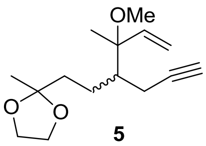

5

A solution of **9** (500 mg, 2.10 mmol) in DMF (2.0 mL) was slowly added to a suspension of sodium hydride (60% in mineral oil, 126 mg, 3.17 mmol) in DMF (3.2 mL) at 0 °C. After stirring for 30 min at this temperature, iodomethane (0.33 mL, 750 mg, 5.3 mmol) was added. The reaction mixture was stirred for 3 h at 0 °C and the reaction quenched with water. The crude mixture was extracted with ether (50 mL × 3). The organic layers were combined, dried over Na_2SO_4, and concentrated. The residue was purified by flash chromatography on silica gel (eluent hexane*:*ethyl acetate = 95:5) to give compound **5** as a colorless oil (470 mg, 1.86 mmol, 88% yield, dr = 3*:*1). R_f = 0.61 (hexane*:*ethyl acetate = 80:20); ^{1}H NMR (300 MHz,

$CDCl_3$): δ = 1.20 (s, 3H), 1.31 (s, 3H), 1.36−1.79 (m, 4H), 1.88−1.98 (m, 2H), 2.16−2.50 (m, 2H), 3.12 (s, 3H), 3.87−3.95 (m, 4H), 5.14 (dd, *J* = 17.4 Hz, *J* = 1.2 Hz, 1H), 5.27 (dd, *J* = 10.8 Hz, *J* = 1.2 Hz, 1H), 5.65 (dd, *J* = 17.4 Hz, *J* = 10.8 Hz, 1H); {distinctive signals for the minor diastereomer} δ = 1.23 (s, 3H), 1.33 (s, 3H), 3.12 (s, 3H), 5.16 (dd, *J* = 17.4 Hz, *J* = 1.2 Hz, 1H), 5.26 (dd, *J* = 10.8 Hz, *J* = 1.2 Hz, 1H), 5.72 (dd, *J* = 17.7 Hz, *J* = 10.8 Hz, 1H); ^{13}C NMR (75 MHz, $CDCl_3$): δ = 16.4, 17.4, 18.2, 18.9, 23.8, 23.8, 23.9, 24.5, 37.9, 38.0, 46.2, 46.3, 50.1, 64.7, 64.8, 69.4, 69.6, 80.1, 84.1, 84.4, 110.3, 110.4, 116.8, 141.2, 141.7; IR: (cm^{-1}) ν 3292, 2982, 1456, 1377, 1218, 1159, 1106, 1063; HRMS calcd for $C_{15}H_{25}O_3$(FAB+): 253.1804. found: 253.1802.

Compound 4

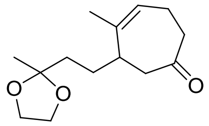

4

A solution of $Au[P(C_6F_5)_3]Cl$ (5.6 mg, 0.0080 mmol) in dry CH_2Cl_2 (1 mL) was added to a solution of $AgSbF_6$ (2.7 mg, 0.0079 mmol) in dry CH_2Cl_2 (1 mL). The solution was stirred for 10 min. The resulting solution was filtered though a pad of Celite and concentrated. The residue was dried under high vacuum for 2 h and then cooled to -15 °C. A solution of compound **5** (100 mg, 0.40 mmol) in dry CH_2Cl_2 (8.0 mL, pre-cooled to -15 °C) was added to this residue. The resulting green solution was stirred for 2 min. Triethylamine (1 mL) was added and the solution stirred for a further 5 min. The resulting solution was filtered through a pad of silica and concentrated. The crude oil was dissolved in CH_2Cl_2 (8.0 mL). Silica gel (150 mg) was added and the reaction mixture was stirred for 15 h. CH_2Cl_2 was removed under reduced pressure. The crude oil was purified by flash chromatography on silica gel (eluent hexane:ethyl acetate = 90:10) to give compound **4** as a colorless oil (89 mg, 0.37 mmol, 93% yield). R_f = 0.28 (hexane:ethyl acetate = 80:20); ^1H NMR (300 MHz, $CDCl_3$): δ = 1.30 (s, 3H), 1.43−1.73 (m, 3H), 1.74 (s, 3H), 1.76−1.80 (m, 1H), 2.14−2.49 (m, 5H), 3.90–3.95 (m, 4H), 5.59−5.64 (m, 1H); ^{13}C NMR (75 MHz, $CDCl_3$): δ = 22.6, 24.0, 25.1, 27.5, 36.4, 41.3, 44.0, 45.8, 64.8, 64.8, 110.0, 124.5, 140.9, 213.1; IR: (cm^{-1}) ν 2956, 2880, 1706, 1450, 1377, 1223, 1067; HRMS calcd for $C_{14}H_{23}O_3$(FAB+): 239.1647. found: 239.1651.

Compound 2

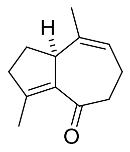

2

Compound **4** (686 mg, 2.30 mmol) was dissolved in a mixture of acetone:water (2/1, 3.3 mL). *p*-TsOH (10 mg) was added. The reaction mixture was stirred for 15 h at rt and the acetone removed under reduced pressure. The crude oil was extracted with diethyl ether (30 mL × 2) and the extracts were washed with sat. $NaHCO_3$ solution. The organic layers were combined, dried over anhydrous Na_2SO_4 and concentrated. The crude mixture was diluted with MeOH (100 mL). Potassium hydroxide (1.29 g, 23.0 mmol) was added, and the resulting solution was heated with stirring at 60 °C for 15 h. MeOH was removed under reduced pressure. The crude oil was extracted with diethyl ether (50 mL × 2), the extracts were washed with sat. NH_4Cl solution (50 mL × 2), dried over anhydrous Na_2SO_4 and concentrated. The residual oil was purified by flash chromatography on silica gel (eluent hexane:ethyl acetate = 95:5) to give compound **2** as a yellow oil (337 mg, 1.91 mmol, 83% yield). R_f = 0.60 (hexane:ethyl acetate = 80:20); ^{1}H NMR (300 MHz, $CDCl_3$): δ = 1.74 (s, 3H), 1.76–1.82 (m, 1H), 2.05 (s, 3H), 2.13 – 2.20 (m, 1H), 2.24 – 2.42 (m, 5H), 2.73–2.79 (m, 1H), 3.70–3.80 (m, 1H), 5.55–5.65 (m, 1H); This spectral data was consistent with the literature values [3]

Compound 3

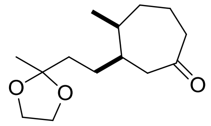

3

The literature procedure [4] was modified. Compound **4** (20 mg, 0.084 mmol) and rhodium/alumina (3.5 mg, 0.0017 mmol) in ethyl acetate (0.42 mL) were stirred for 3 h at 0 °C under a hydrogen atmosphere (balloon). The reaction mixture was filtered through a pad of Celite and concentrated. The residue was purified by flash chromatography on silica gel (eluent hexane:ethyl acetate = 90:10) to give compound 3 as a colorless oil (19 mg, 0.079 mmol, 94% yield, cis:trans = 13:1). R_f = 0.31 (hexane:ethyl acetate = 70:30); The spectral data of the major compound was consistent with the literature data [4]; ^{1}H NMR (300 MHz, $CDCl_3$): δ = 0.87 (d, *J* = 7.2 Hz, 3H, for cis-isomer), 0.99 (d, *J* = 6.6 Hz, 3H, for trans-isomer), 1.22–1.28 (m, 1H), 1.30 (s, 3H), 1.38–1.75 (m, 7H), 1.77–1.86 (m, 1H), 1.94–2.00 (m, 1H), 2.31–2.53 (m, 4H), 3.89–3.95 (m, 4H); ^{13}C NMR (75 MHz, $CDCl_3$): δ = 14.7, 20.8, 23.9, 27.2, 35.8, 36.9, 37.3, 39.3, 44.0, 46.3, 64.8, 64.8, 110.2, 214.6.

References:

1. Shin, S. *Bull. Kor. Chem. Soc.* **2005**, *26*, 1925.
2. Rosowsky, A.; Mota, C. E.; Wright, J. E.; Freisheim, J. H.; Heusner, J. J.; McCormack, J. J.; Queener, S. F. *J. Med. Chem.* **1993**, *36*, 3103.
3. Sampath, V.; Lund, E. C.; Knudsen, M. J.; Olmstead, M. M.; Schore, N. E. *J. Org. Chem.* **1987**, *52*, 3595.
4. Kim, S. K.; Pak, C. S. *J. Org. Chem.* **1991**, *56*, 6829.

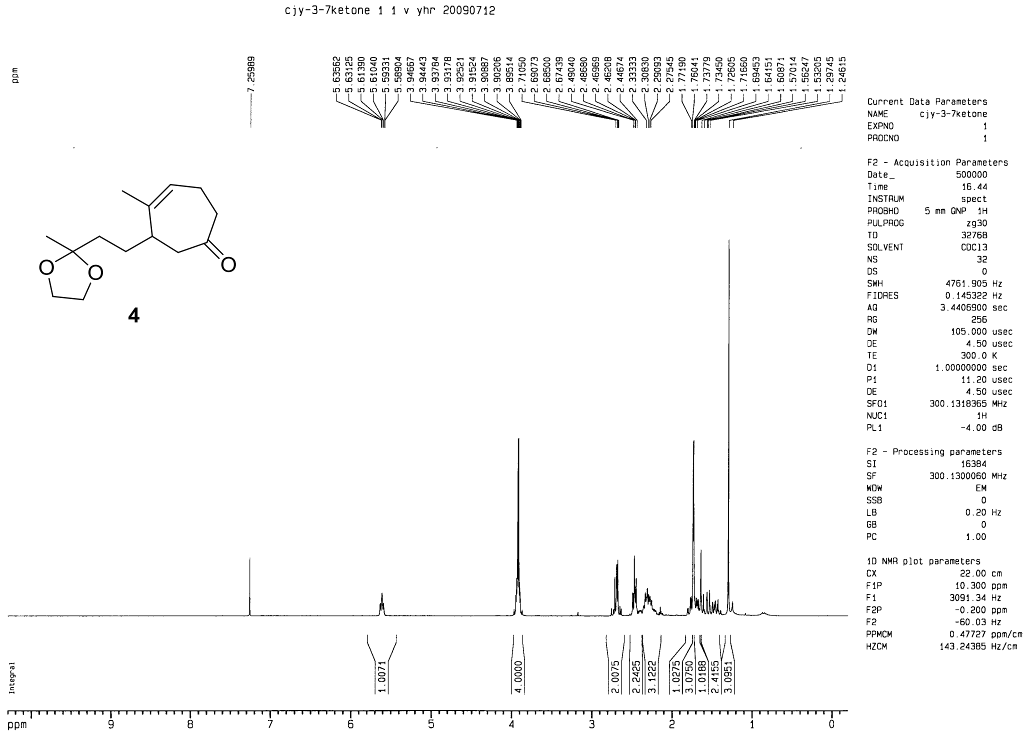
cjy-3-7ketone 1 1 v yhr 20090712
ppm
7.25989
5.63562
5.63125
5.61390
5.61040
5.59931
5.58904
3.94667
3.94443
3.93784
3.93178
3.92521
3.91524
3.90887
3.90206
3.89514
2.71050
2.69073
2.68500
2.67439
2.49040
2.48680
2.46969
2.46208
2.44674
2.33333
2.30830
2.29093
2.27545
1.77190
1.76041
1.73779
1.73450
1.72605
1.71660
1.69453
1.64151
1.60871
1.57014
1.56247
1.53205
1.29745
1.24615
4
Integral
1.0071
4.0000
2.0075
2.2425
3.1222
1.0275
3.0750
1.0188
2.4155
3.0951
ppm 9 8 7 6 5 4 3 2 1 0
Current Data Parameters
NAME cjy-3-7ketone
EXPNO 1
PROCNO 1
F2 - Acquisition Parameters
Date_ 500000
Time 16.44
INSTRUM spect
PROBHD 5 mm QNP 1H
PULPROG zg30
TD 32768
SOLVENT CDCl3
NS 32
DS 0
SWH 4761.905 Hz
FIDRES 0.145322 Hz
AQ 3.4406900 sec
RG 256
DW 105.000 usec
DE 4.50 usec
TE 300.0 K
D1 1.00000000 sec
P1 11.20 usec
DE 4.50 usec
SFO1 300.1318365 MHz
NUC1 1H
PL1 -4.00 dB
F2 - Processing parameters
SI 16384
SF 300.1300060 MHz
WDW EM
SSB 0
LB 0.20 Hz
GB 0
PC 1.00
1D NMR plot parameters
CX 22.00 cm
F1P 10.300 ppm
F1 3091.34 Hz
F2P -0.200 ppm
F2 -60.03 Hz
PPMCM 0.47727 ppm/cm
HZCM 143.24385 Hz/cm

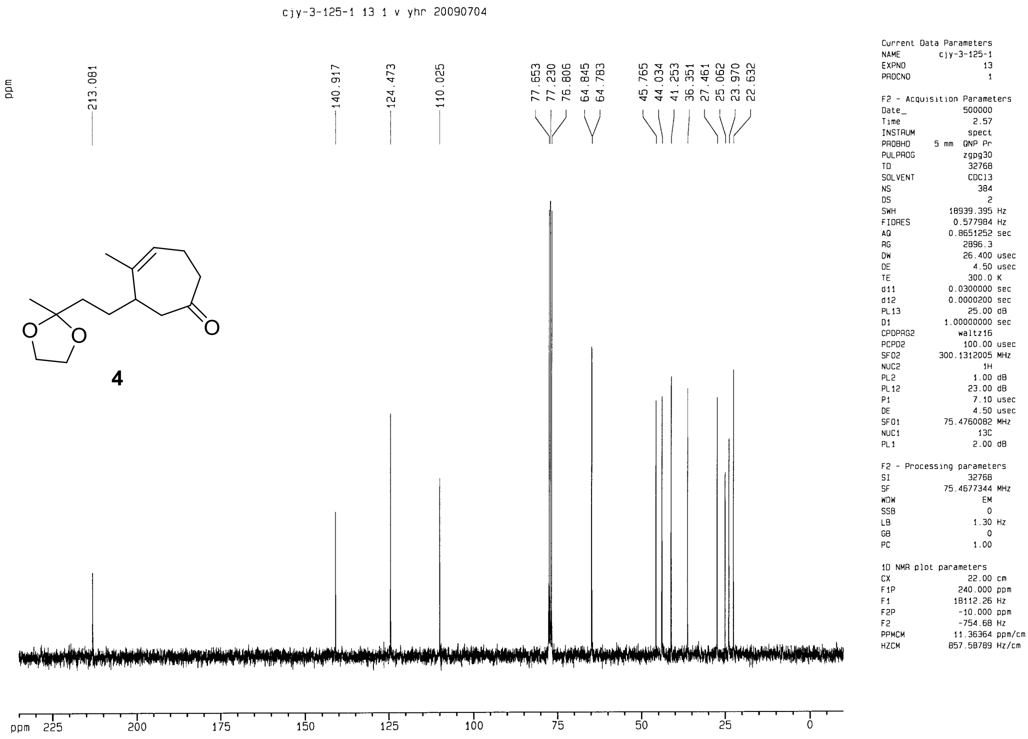
cjy-3-125-1 13 1 v yhr 20090704
ppm
213.081
140.917
124.473
110.025
77.653
77.230
76.806
64.845
64.783
45.765
44.034
41.253
36.351
27.461
25.062
23.970
22.632
4
ppm 225 200 175 150 125 100 75 50 25 0
Current Data Parameters
NAME cjy-3-125-1
EXPNO 13
PROCNO 1
F2 - Acquisition Parameters
Date_ 500000
Time 2.57
INSTRUM spect
PROBHD 5 mm QNP Pr
PULPROG zgpg30
TD 32768
SOLVENT CDCl3
NS 384
DS 2
SWH 18939.395 Hz
FIDRES 0.577984 Hz
AQ 0.8651252 sec
RG 2896.3
DW 26.400 usec
DE 4.50 usec
TE 300.0 K
d11 0.0300000 sec
d12 0.0000200 sec
PL13 25.00 dB
D1 1.00000000 sec
CPDPRG2 waltz16
PCPD2 100.00 usec
SFO2 300.1312005 MHz
NUC2 1H
PL2 1.00 dB
PL12 23.00 dB
P1 7.10 usec
DE 4.50 usec
SFO1 75.4760082 MHz
NUC1 13C
PL1 2.00 dB
F2 - Processing parameters
SI 32768
SF 75.4677344 MHz
WDW EM
SSB 0
LB 1.30 Hz
GB 0
PC 1.00
1D NMR plot parameters
CX 22.00 cm
F1P 240.000 ppm
F1 18112.26 Hz
F2P -10.000 ppm
F2 -754.68 Hz
PPMCM 11.36364 ppm/cm
HZCM 857.58789 Hz/cm

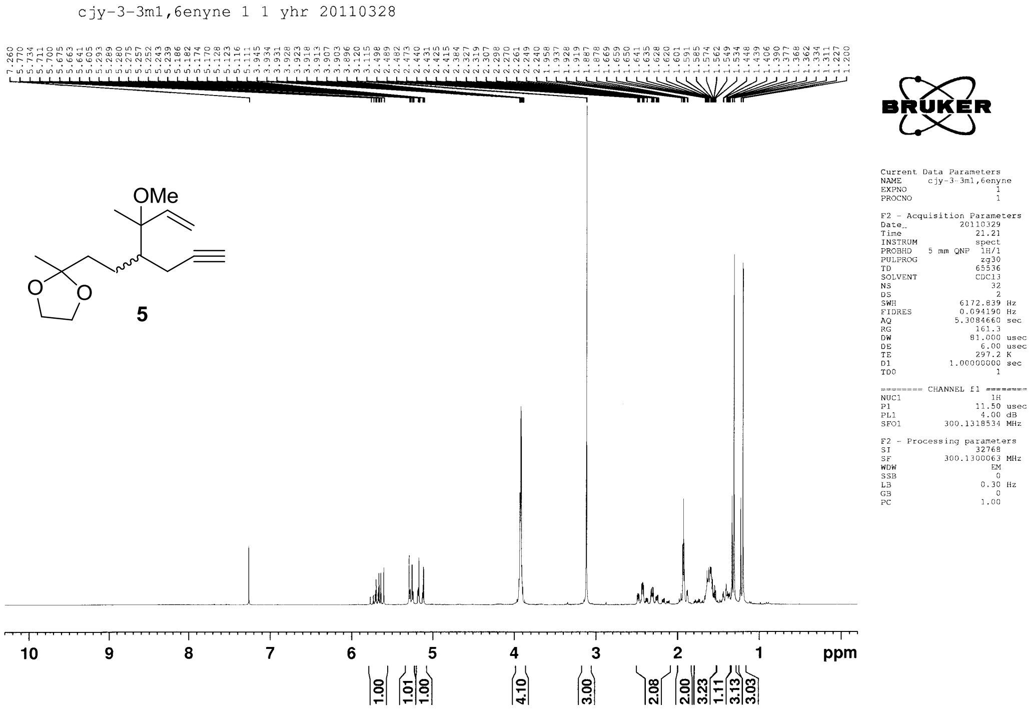
cjy-3-3m1,6enyne 1 1 yhr 20110328
OMe
5
ppm
Current Data Parameters
NAME cjy-3-3m1,6enyne
EXPNO 1
PROCNO 1
F2 - Acquisition Parameters
Date_ 20110329
Time 21.21
INSTRUM spect
PROBHD 5 mm QNP 1H/1
PULPROG zg30
TD 65536
SOLVENT CDCl3
NS 32
DS 2
SWH 6172.839 Hz
FIDRES 0.094190 Hz
AQ 5.3084660 sec
RG 161.3
DW 81.000 usec
DE 6.00 usec
TE 297.2 K
D1 1.00000000 sec
TD0 1
======== CHANNEL f1 ========
NUC1 1H
P1 11.50 usec
PL1 4.00 dB
SFO1 300.1318534 MHz
F2 - Processing parameters
SI 32768
SF 300.1300063 MHz
WDW EM
SSB 0
LB 0.30 Hz
GB 0
PC 1.00

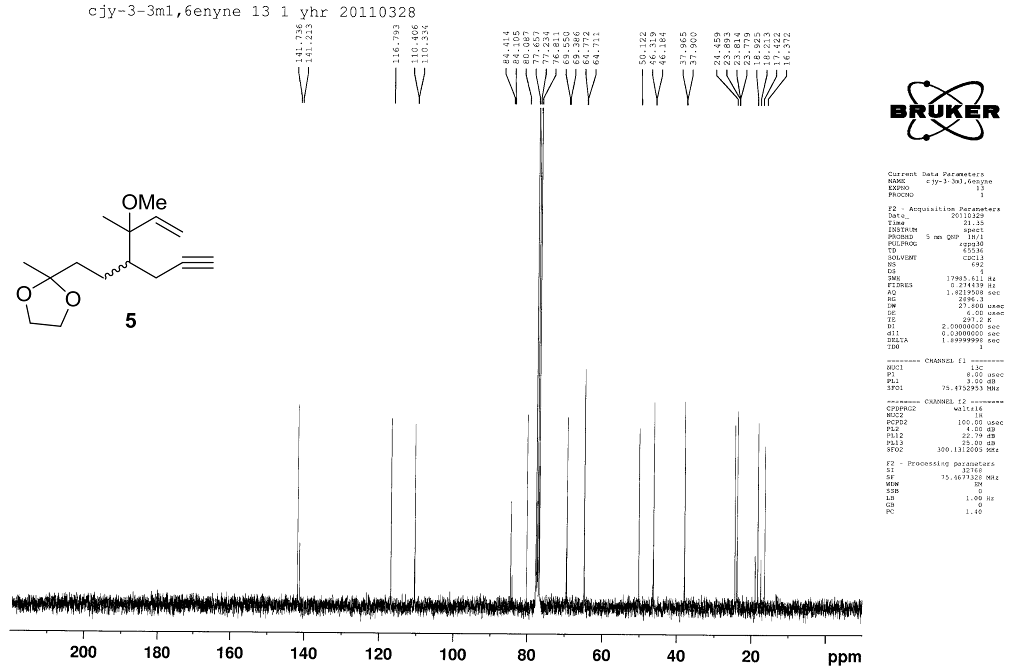
cjy-3-3m1,6enyne 13 1 yhr 20110328
141.736
141.213
116.793
110.406
110.334
84.414
84.105
80.087
77.657
77.234
76.811
69.550
69.386
64.772
64.711
50.122
46.319
46.184
37.965
37.900
24.459
23.893
23.814
23.779
18.925
18.213
17.422
16.372
OMe
5
200
180
160
140
120
100
80
60
40
20
ppm

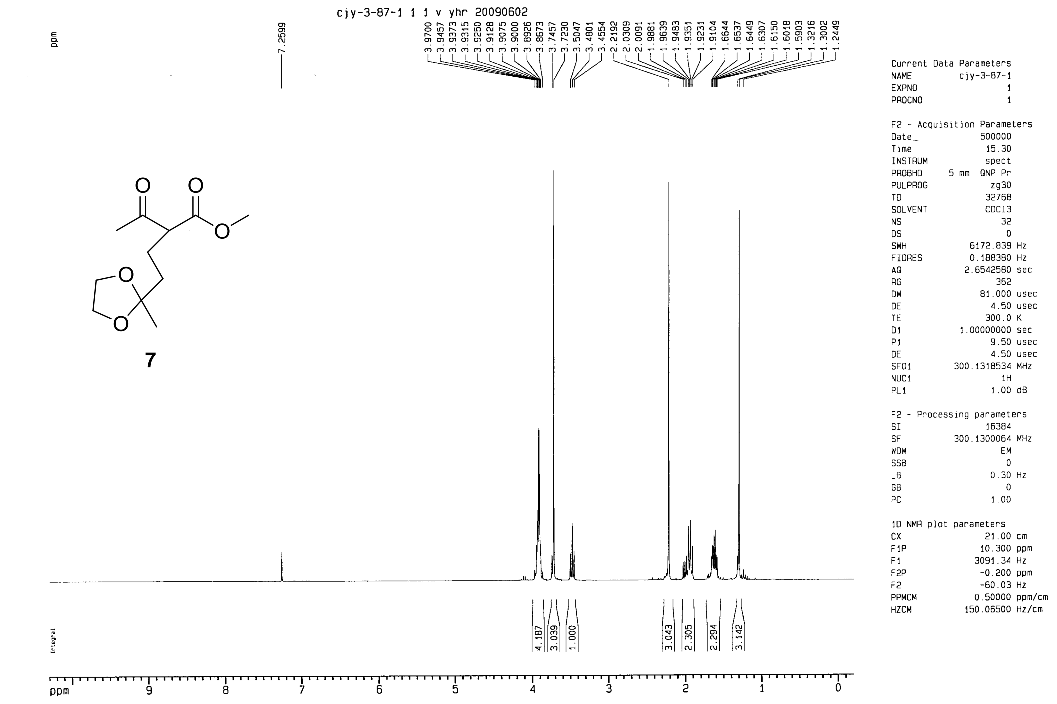
cjy-3-87-1 1 1 v yhr 20090602
7.2599
3.9700 3.9457 3.9373 3.9315 3.9250 3.9128 3.9075 3.9000 3.8926 3.8673 3.7457 3.7230 3.5047 3.4801 3.4554 2.2192 2.0309 2.0091 1.9881 1.9639 1.9483 1.9351 1.9231 1.9104 1.6644 1.6537 1.6449 1.6307 1.6150 1.6018 1.5903 1.3216 1.3002 1.2449
7
4.187 3.039 1.000 3.043 2.305 2.294 3.142
ppm 9 8 7 6 5 4 3 2 1 0
Current Data Parameters
NAME cjy-3-87-1
EXPNO 1
PROCNO 1
F2 - Acquisition Parameters
Date_ 500000
Time 15.30
INSTRUM spect
PROBHD 5 mm QNP Pr
PULPROG zg30
TD 32768
SOLVENT CDCl3
NS 32
DS 0
SWH 6172.839 Hz
FIDRES 0.188380 Hz
AQ 2.6542580 sec
RG 362
DW 81.000 usec
DE 4.50 usec
TE 300.0 K
D1 1.00000000 sec
P1 9.50 usec
DE 4.50 usec
SFO1 300.1318534 MHz
NUC1 1H
PL1 1.00 dB
F2 - Processing parameters
SI 16384
SF 300.1300064 MHz
WDW EM
SSB 0
LB 0.30 Hz
GB 0
PC 1.00
1D NMR plot parameters
CX 21.00 cm
F1P 10.300 ppm
F1 3091.34 Hz
F2P -0.200 ppm
F2 -60.03 Hz
PPMCM 0.50000 ppm/cm
HZCM 150.06500 Hz/cm

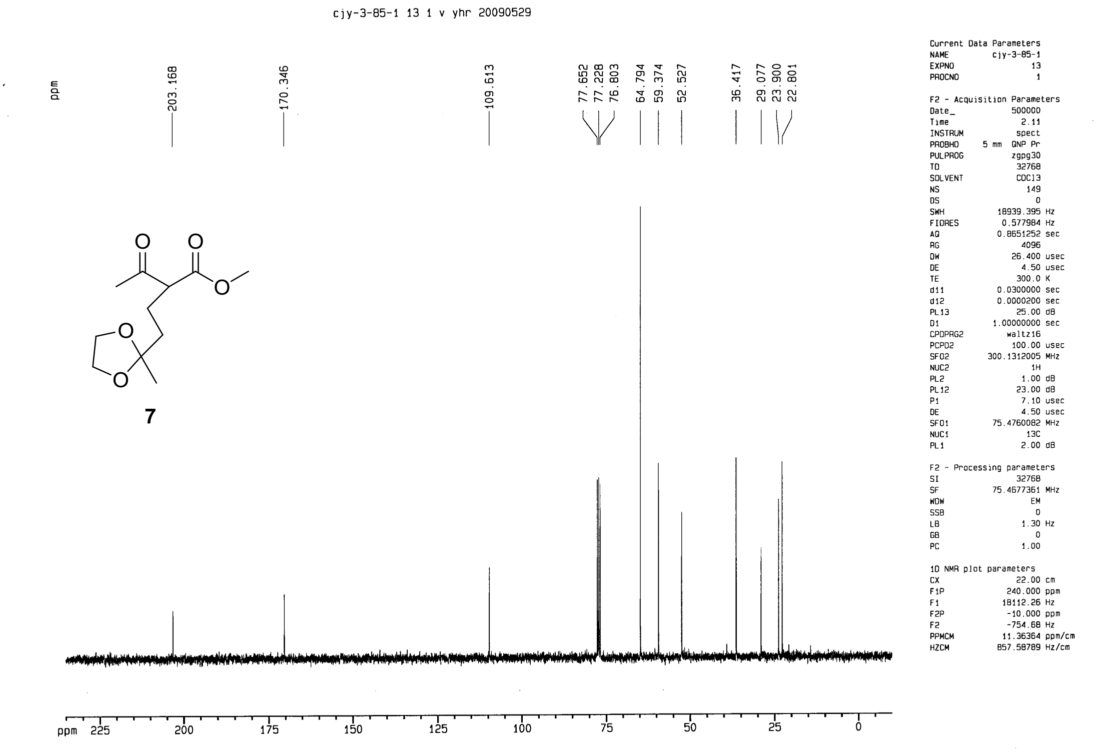
cjy-3-85-1 13 1 v yhr 20090529
203.168 170.346 109.613 77.652 77.228 76.803 64.794 59.374 52.527 36.417 29.077 23.900 22.801
7
ppm 225 200 175 150 125 100 75 50 25 0
Current Data Parameters
NAME cjy-3-85-1
EXPNO 13
PROCNO 1
F2 - Acquisition Parameters
Date_ 500000
Time 2.11
INSTRUM spect
PROBHD 5 mm QNP Pr
PULPROG zgpg30
TD 32768
SOLVENT CDCl3
NS 149
DS 0
SWH 18939.395 Hz
FIDRES 0.577984 Hz
AQ 0.8651252 sec
RG 4096
DW 26.400 usec
DE 4.50 usec
TE 300.0 K
d11 0.0300000 sec
d12 0.0000200 sec
PL13 25.00 dB
D1 1.00000000 sec
CPDPRG2 waltz16
PCPD2 100.00 usec
SFO2 300.1312005 MHz
NUC2 1H
PL2 1.00 dB
PL12 23.00 dB
P1 7.10 usec
DE 4.50 usec
SFO1 75.4760082 MHz
NUC1 13C
PL1 2.00 dB
F2 - Processing parameters
SI 32768
SF 75.4677361 MHz
WDW EM
SSB 0
LB 1.30 Hz
GB 0
PC 1.00
1D NMR plot parameters
CX 22.00 cm
F1P 240.000 ppm
F1 18112.26 Hz
F2P -10.000 ppm
F2 -754.68 Hz
PPMCM 11.36364 ppm/cm
HZCM 857.58789 Hz/cm

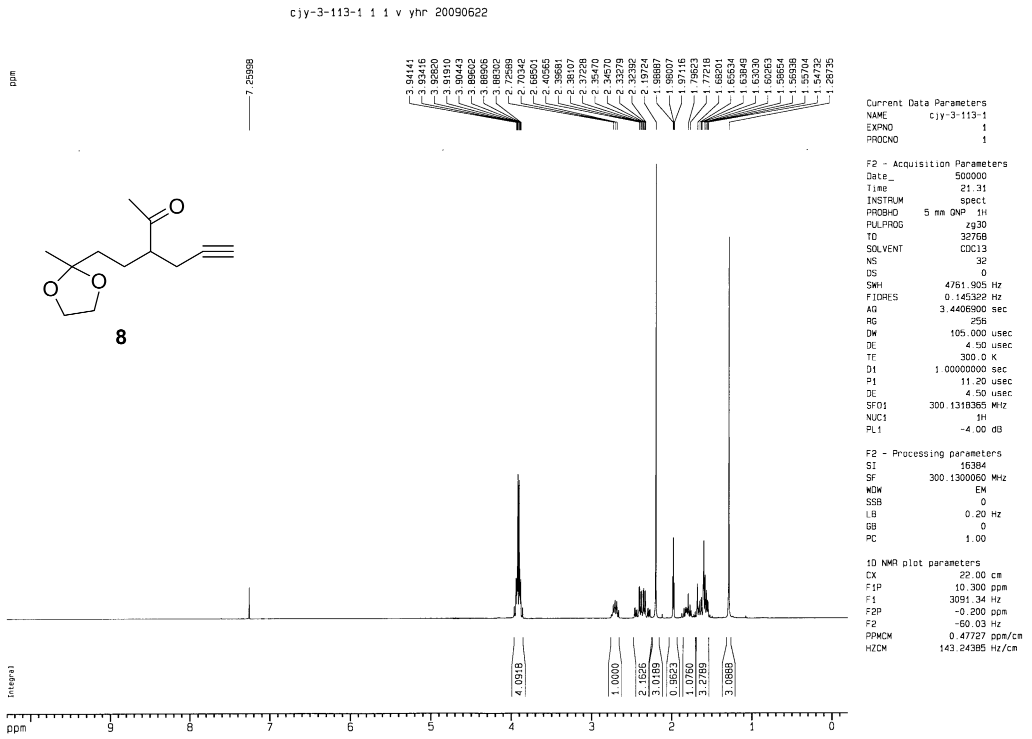
cjy-3-113-1 1 1 v yhr 20090622
7.25998
3.94141 3.93416 3.92820 3.91910 3.90443 3.89602 3.88906 3.88302 3.72589 2.70342 2.68501 2.40565 2.39681 2.38107 2.37228 2.35470 2.34570 2.33279 2.32392 2.19724 1.98887 1.98007 1.97116 1.79623 1.77218 1.68201 1.65634 1.63849 1.63030 1.60263 1.58654 1.56938 1.55704 1.54732 1.28735
8
4.0918
1.0000 2.1626 3.0189 0.9623 1.0760 3.2789 3.0888
ppm 9 8 7 6 5 4 3 2 1 0
Current Data Parameters
NAME cjy-3-113-1
EXPNO 1
PROCNO 1
F2 - Acquisition Parameters
Date_ 500000
Time 21.31
INSTRUM spect
PROBHD 5 mm QNP 1H
PULPROG zg30
TD 32768
SOLVENT CDCl3
NS 32
DS 0
SWH 4761.905 Hz
FIDRES 0.145322 Hz
AQ 3.4406900 sec
RG 256
DW 105.000 usec
DE 4.50 usec
TE 300.0 K
D1 1.00000000 sec
P1 11.20 usec
DE 4.50 usec
SFO1 300.1318365 MHz
NUC1 1H
PL1 -4.00 dB
F2 - Processing parameters
SI 16384
SF 300.1300060 MHz
WDW EM
SSB 0
LB 0.20 Hz
GB 0
PC 1.00
1D NMR plot parameters
CX 22.00 cm
F1P 10.300 ppm
F1 3091.34 Hz
F2P -0.200 ppm
F2 -60.03 Hz
PPMCM 0.47727 ppm/cm
HZCM 143.24385 Hz/cm

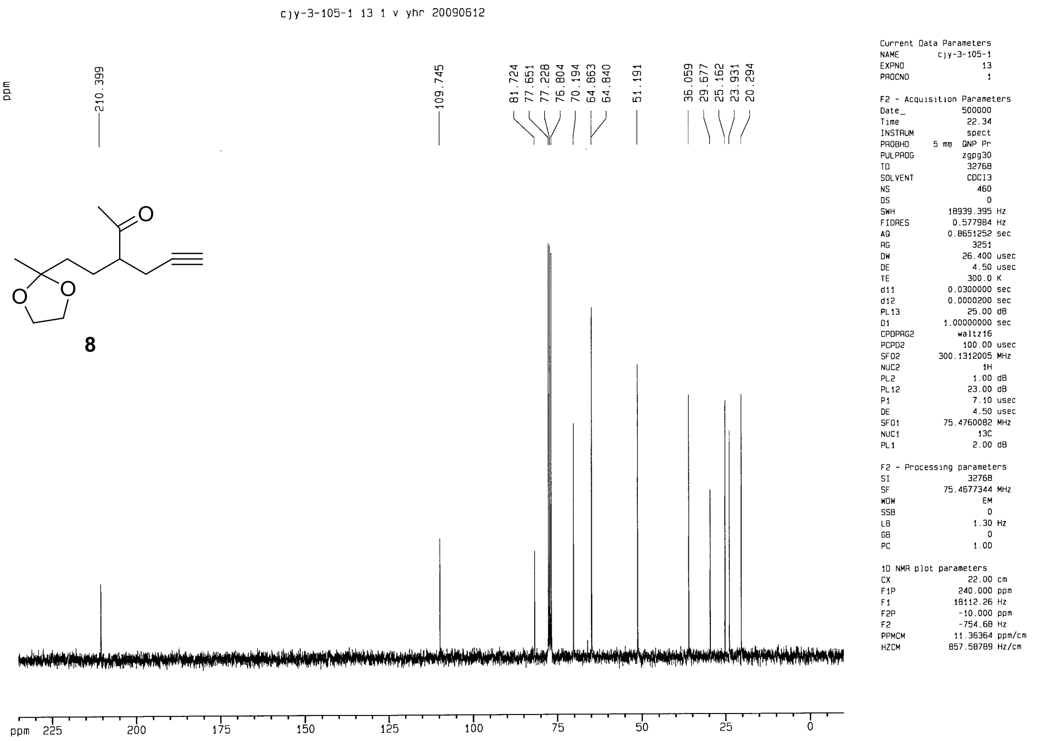
cjy-3-105-1 13 1 v yhr 20090612
210.399
109.745
81.724 77.651 77.228 76.804 70.194 64.863 64.840
51.191
36.059 29.677 25.162 23.931 20.294
8
ppm 225 200 175 150 125 100 75 50 25 0
Current Data Parameters
NAME cjy-3-105-1
EXPNO 13
PROCNO 1
F2 - Acquisition Parameters
Date_ 500000
Time 22.34
INSTRUM spect
PROBHD 5 mm QNP Pr
PULPROG zgpg30
TD 32768
SOLVENT CDCl3
NS 460
DS 0
SWH 18939.395 Hz
FIDRES 0.577984 Hz
AQ 0.8651252 sec
RG 3251
DW 26.400 usec
DE 4.50 usec
TE 300.0 K
d11 0.0300000 sec
d12 0.0000200 sec
PL13 25.00 dB
D1 1.00000000 sec
CPDPRG2 waltz16
PCPD2 100.00 usec
SFO2 300.1312005 MHz
NUC2 1H
PL2 1.00 dB
PL12 23.00 dB
P1 7.10 usec
DE 4.50 usec
SFO1 75.4760082 MHz
NUC1 13C
PL1 2.00 dB
F2 - Processing parameters
SI 32768
SF 75.4677344 MHz
WDW EM
SSB 0
LB 1.30 Hz
GB 0
PC 1.00
1D NMR plot parameters
CX 22.00 cm
F1P 240.000 ppm
F1 18112.26 Hz
F2P -10.000 ppm
F2 -754.68 Hz
PPMCM 11.36364 ppm/cm
HZCM 857.58789 Hz/cm

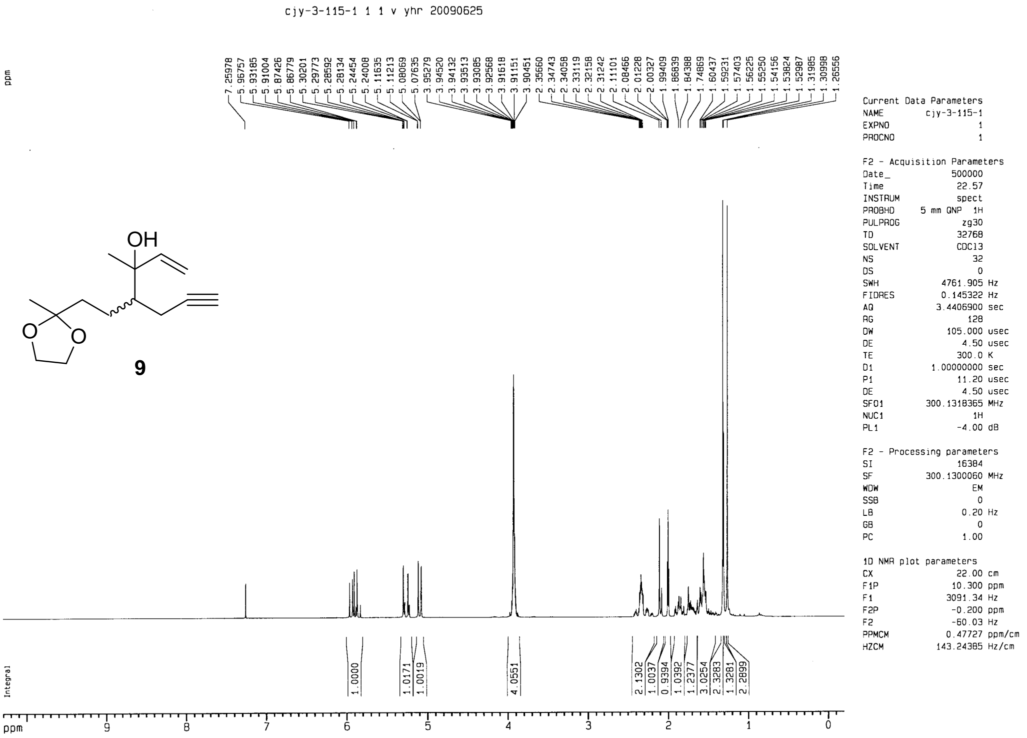
cjy-3-115-1 1 1 v yhr 20090625
7.25978
5.96757
5.93185
5.91004
5.87426
5.86779
5.30201
5.29773
5.28592
5.28134
5.24454
5.24008
5.11635
5.11213
5.08069
5.07635
3.95279
3.94520
3.94132
3.93513
3.93085
3.92568
3.91618
3.91151
3.90451
2.35660
2.34743
2.34058
2.33119
2.32158
2.31242
2.11101
2.08466
2.01228
2.00327
1.99409
1.86839
1.84388
1.74869
1.60437
1.59231
1.57403
1.56225
1.55250
1.54156
1.53824
1.52987
1.31985
1.30998
1.26556
OH
O
O
9
1.0000
1.0171
1.0019
4.0551
2.1302
1.0037
0.9394
1.0392
1.2377
3.0254
2.3283
1.3281
2.2899
Integral
ppm 9 8 7 6 5 4 3 2 1 0
Current Data Parameters
NAME cjy-3-115-1
EXPNO 1
PROCNO 1
F2 - Acquisition Parameters
Date_ 500000
Time 22.57
INSTRUM spect
PROBHD 5 mm QNP 1H
PULPROG zg30
TD 32768
SOLVENT CDCl3
NS 32
DS 0
SWH 4761.905 Hz
FIDRES 0.145322 Hz
AQ 3.4406900 sec
RG 128
DW 105.000 usec
DE 4.50 usec
TE 300.0 K
D1 1.00000000 sec
P1 11.20 usec
DE 4.50 usec
SFO1 300.1318365 MHz
NUC1 1H
PL1 -4.00 dB
F2 - Processing parameters
SI 16384
SF 300.1300060 MHz
WDW EM
SSB 0
LB 0.20 Hz
GB 0
PC 1.00
1D NMR plot parameters
CX 22.00 cm
F1P 10.300 ppm
F1 3091.34 Hz
F2P -0.200 ppm
F2 -60.03 Hz
PPMCM 0.47727 ppm/cm
HZCM 143.24385 Hz/cm

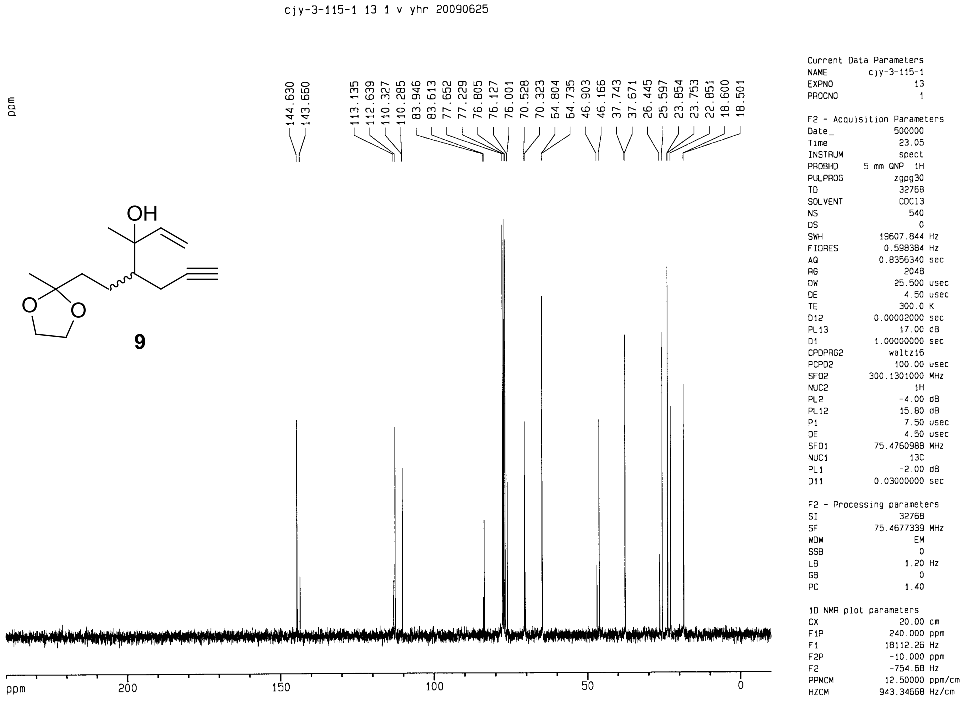
cjy-3-115-1 13 1 v yhr 20090625
144.630
143.660
113.135
112.639
110.327
110.285
83.946
83.613
77.652
77.229
76.805
76.127
76.001
70.528
70.323
64.804
64.735
46.903
46.166
37.743
37.671
26.445
25.597
23.854
23.753
22.851
18.600
18.501
OH
O
O
9
ppm 200 150 100 50 0
Current Data Parameters
NAME cjy-3-115-1
EXPNO 13
PROCNO 1
F2 - Acquisition Parameters
Date_ 500000
Time 23.05
INSTRUM spect
PROBHD 5 mm QNP 1H
PULPROG zgpg30
TD 32768
SOLVENT CDCl3
NS 540
DS 0
SWH 19607.844 Hz
FIDRES 0.598384 Hz
AQ 0.8356340 sec
RG 2048
DW 25.500 usec
DE 4.50 usec
TE 300.0 K
D12 0.00002000 sec
PL13 17.00 dB
D1 1.00000000 sec
CPDPRG2 waltz16
PCPD2 100.00 usec
SFO2 300.1301000 MHz
NUC2 1H
PL2 -4.00 dB
PL12 15.80 dB
P1 7.50 usec
DE 4.50 usec
SFO1 75.4760988 MHz
NUC1 13C
PL1 -2.00 dB
D11 0.03000000 sec
F2 - Processing parameters
SI 32768
SF 75.4677339 MHz
WDW EM
SSB 0
LB 1.20 Hz
GB 0
PC 1.40
1D NMR plot parameters
CX 20.00 cm
F1P 240.000 ppm
F1 18112.26 Hz
F2P -10.000 ppm
F2 -754.68 Hz
PPMCM 12.50000 ppm/cm
HZCM 943.34668 Hz/cm

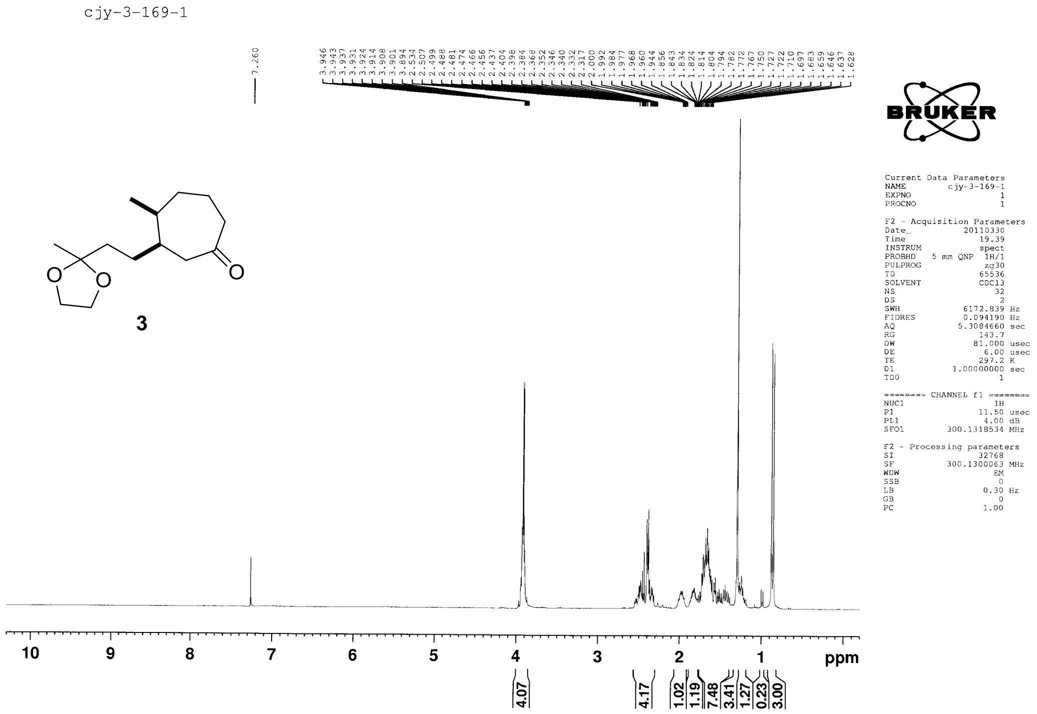
cjy-3-169-1
7.260
3.946 3.943 3.937 3.931 3.924 3.914 3.908 3.901 3.894 2.534 2.507 2.499 2.488 2.481 2.474 2.466 2.456 2.437 2.404 2.398 2.384 2.368 2.352 2.346 2.340 2.332 2.317 2.000 1.992 1.984 1.977 1.968 1.960 1.944 1.856 1.843 1.834 1.824 1.814 1.804 1.794 1.782 1.772 1.767 1.750 1.727 1.722 1.710 1.697 1.683 1.659 1.646 1.637 1.628
O
O
O
3
BRUKER
Current Data Parameters
NAME cjy-3-169-1
EXPNO 1
PROCNO 1
F2 - Acquisition Parameters
Date_ 20110330
Time 19.39
INSTRUM spect
PROBHD 5 mm QNP 1H/1
PULPROG zg30
TD 65536
SOLVENT CDCl3
NS 32
DS 2
SWH 6172.839 Hz
FIDRES 0.094190 Hz
AQ 5.3084660 sec
RG 143.7
DW 81.000 usec
DE 6.00 usec
TE 297.2 K
D1 1.00000000 sec
TD0 1
======== CHANNEL f1 ========
NUC1 1H
P1 11.50 usec
PL1 4.00 dB
SFO1 300.1318534 MHz
F2 - Processing parameters
SI 32768
SF 300.1300063 MHz
WDW EM
SSB 0
LB 0.30 Hz
GB 0
PC 1.00
10 9 8 7 6 5 4 3 2 1 ppm
4.07
4.17
1.02
1.19
7.48
3.41
1.27
0.23
3.00

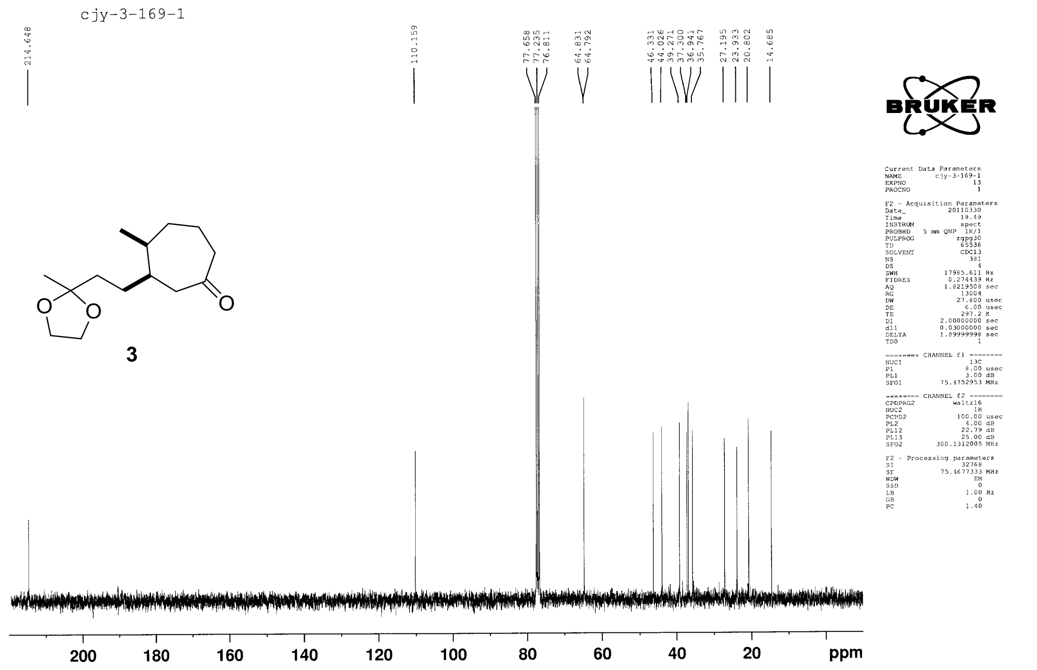
cjy-3-169-1
214.648
110.159
77.658 77.235 76.811
64.831 64.792
46.331 44.026 39.271 37.300 36.941 35.767
27.195 23.933 20.802
14.685
O
O
O
3
BRUKER
Current Data Parameters
NAME cjy-3-169-1
EXPNO 13
PROCNO 1
F2 - Acquisition Parameters
Date_ 20110330
Time 19.49
INSTRUM spect
PROBHD 5 mm QNP 1H/1
PULPROG zgpg30
TD 65536
SOLVENT CDCl3
NS 381
DS 4
SWH 17985.611 Hz
FIDRES 0.274439 Hz
AQ 1.8219508 sec
RG 13004
DW 27.800 usec
DE 6.00 usec
TE 297.2 K
D1 2.00000000 sec
d11 0.03000000 sec
DELTA 1.89999998 sec
TD0 1
======== CHANNEL f1 ========
NUC1 13C
P1 8.00 usec
PL1 3.00 dB
SFO1 75.4752953 MHz
======== CHANNEL f2 ========
CPDPRG2 waltz16
NUC2 1H
PCPD2 100.00 usec
PL2 4.00 dB
PL12 22.79 dB
PL13 25.00 dB
SFO2 300.1312005 MHz
F2 - Processing parameters
SI 32768
SF 75.4677333 MHz
WDW EM
SSB 0
LB 1.00 Hz
GB 0
PC 1.40
200 180 160 140 120 100 80 60 40 20 ppm
